# Tumor cells rely on the thiol oxidoreductase PDI for PERK signaling in order to survive ER stress

**DOI:** 10.1038/s41598-020-72259-1

**Published:** 2020-09-17

**Authors:** Philip Kranz, Christopher Sänger, Alexandra Wolf, Jennifer Baumann, Eric Metzen, Melanie Baumann, Kirsten Göpelt, Ulf Brockmeier

**Affiliations:** 1 Institut für Physiologie, Universität Duisburg-Essen, Duisburg, Germany; 2 Department of Neurology, University Hospital Essen, University Duisburg-Essen, Duisburg, Germany

**Keywords:** Cancer, Cell biology

## Abstract

Upon ER stress cells activate the unfolded protein response through PERK, IRE1 and ATF6. Remarkable effort has been made to delineate the downstream signaling of these three ER stress sensors after activation, but upstream regulation at the ER luminal site still remains mostly undefined. Here we report that the thiol oxidoreductase PDI is mandatory for activation of the PERK pathway in HEK293T as well as in human pancreatic, lung and colon cancer cells. Under ER stress, depletion of PDI selectively abrogated eIF2α phosphorylation, induction of ATF4, CHOP and even BiP. Furthermore, we could demonstrate that PDI prevented degradation of activated PERK by the 26S proteasome and therefore contributes to maintained PERK signaling. As a result of decreased PERK activity, PDI depleted cells showed an increased vulnerability to ER stress induced by chemicals or ionizing radiation in 2D as well as in 3D culture models. We conclude that PDI is an obligatory regulator of the PERK pathway with future therapy implications.

## Introduction

The endoplasmic reticulum (ER) acts as the major calcium storage site in eukaryotic cells and is indispensable for steroid hormone and lipid synthesis^1^. Furthermore, protein synthesis and protein folding of secretory and membrane proteins take place at the rough ER. Depending on the origin of the cell and its specific function, 30 up to 50% of all cellular proteins pass this organelle to achieve disulfide bonding and proper folding^2,3^. This is executed through the highly oxidative environment in the ER and supported by classes of specialized enzymes like chaperones and ER oxidoreductases^4^. The capacities of this organelle are easily exceeded by intrinsic (genetic aberrations, ROS, high demand of secretory proteins, cell division etc.) and extrinsic factors (hypoxia, chemo- and radiotherapy, lack of nutrients etc.) which then result in unfolded protein burden and ER stress^5^. In these situations, cells are able to activate an evolutionary conserved program named the unfolded protein response (UPR). The UPR is carried out through the three sensor proteins the inositol-requiring enzyme 1 (IRE1), the activating transcription factor 6 (ATF6) and the protein kinase R (PKR)-like endoplasmic reticulum kinase (PERK), all of them trying to decrease protein burden at the ER and increasing the folding capacity by upregulating specific UPR target genes^6^. Downstream signaling of IRE1, ATF6 and PERK has been studied extensively throughout the past decades, but how these three sensors are activated from the luminal site of the ER is still under investigation^7^. So far, some members of the protein disulfide isomerase family (PDIs) have been identified as regulators for ATF6, IRE1 and in parts even for PERK^8–12^.

In the competition model, it is thought that upon ER stress, PERK is released from binding immunoglobulin protein (BiP), forms dimers and oligomers which are then able to autophosphorylate themselves^7^. PERK then transmits a signal to the cytoplasm by phosphorylation of translation initiation factor-2α (eIF2α), which leads to an immediate translational repression to prevent further unfolded protein burden in the ER. Cap independent translation is increased in return and results in elevated translation of activating transcription factor 4 (ATF4). ATF4 upregulates a set of UPR targets genes like GRP94, BiP and PDI to increase the folding capacity and cope with the stress. After longer exposure to ER stress, ATF4 is responsible for an increase in proapoptotic C/EBP-homologous protein (CHOP) expression and therefore contributes to cell death observed under chronic ER stress^6^.

PERK has been shown to support tumor growth, metastasis, autophagy and radiation resistance and was therefore proposed as a future therapy target to overcome therapy failure^13–20^. Small molecule inhibitors were designed to inhibit PERK phosphorylation and its downstream signaling. In preclinical studies they were successfully tested in antitumor treatment but showed severe side effects^21–23^. Therefore, identifying new necessary upstream regulators of PERK at the ER luminal side could be an opportunity to limit PERK activity in tumors while sparing adverse effects. PDIA1 (afterwards referred to as PDI) is one of the most abundant proteins in the ER and participates in disulfide bond reduction, oxidation and isomerization. Although the disulfide bonding function of PDI has been shown in numerous studies^24,25^, client proteins which rely on PDI’s function are rare and loss of PDI can be compensated by other PDI family members (e.g. ERp57 or Erp46)^26–28^. These observations presume further functions in regulating ER homeostasis or other undiscovered cellular roles for PDI. Previously, we could show in a colorectal cancer cell model, that upon depletion of the thiol oxidoreductase ERp57, PERK gets activated in a PDI dependent manner and reduces proliferation, induces cell death and sensitizes cancer cells to ionizing radiation^29,30^. Thus, PDI represents a promising target to overcome tumor cell resistance. Here we demonstrate the general validity of a PDI dependency for PERK signaling during acute and prolonged ER stress in a set of various human cancer cell lines. We also expand PDI’s role as a PERK activator to that of a maintainer of PERK signaling and thus offer a new therapeutic strategy to inhibit PERK signaling in tumor cells.

## Results

### PDI is mandatory for activation of the PERK pathway during acute and chronic ER stress

**Figure 1:**
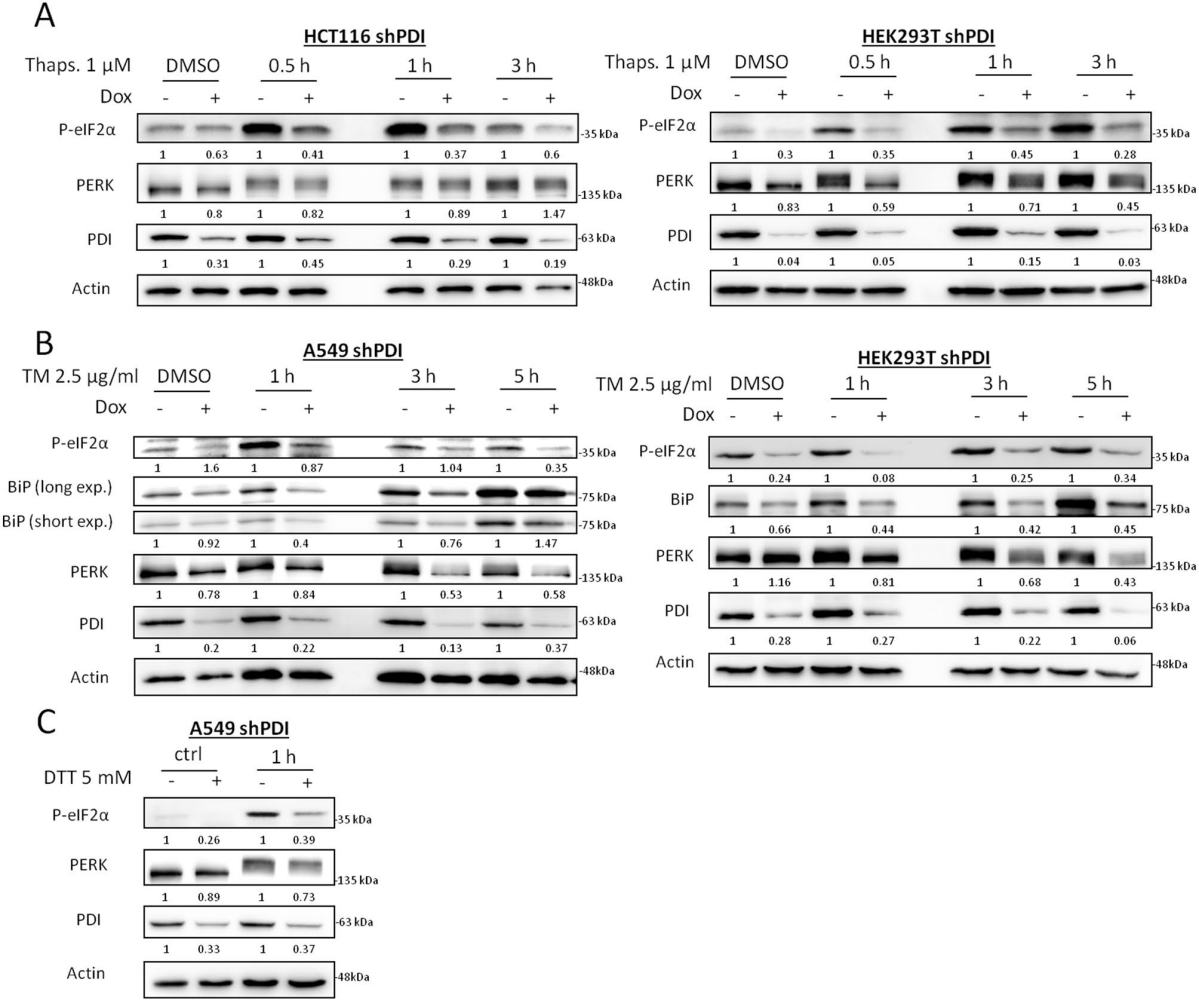
PDI is mandatory for PERK activation under acute ER stress. **(A)** HCT116 and HEK293T shPDI cells were treated with DMSO or 1 µM thapsigargin (Thaps.) for 0.5, 1 and 3 h after 72 h of KD induction. Expression of P-eIF2α, PERK, PDI and Actin were analyzed by western blotting. **(B)** A549 and HEK293T shPDI cells were cultured in the presence of DMSO or 2.5 µg/ml tunicamycin (TM) for 1, 3 and 5 h after 72 h of KD induction. Protein expression of P-eIF2α, BiP, PERK, PDI and Actin were tested by western blot analysis. **(C)** A549 shPDI cells were cultured in the presence or absence of 5 mM DTT for 1 h after 72 h of KD induction. Phosphorylation of eIF2α, expression of PERK, PDI and Actin were analyzed by western blotting. In all subfigures, densitometry was used to quantify protein band intensity. After normalization to Actin, the induced KD sample (+ Dox) was compared to its control sample (− Dox, set to 1).

To assess the importance of PDI for PERK activation under ER stress, HCT116, HEK293T, A549 and BxPC3 cells harboring a stable inducible shRNA to knockdown (KD) PDI were generated by lentiviral transduction. Since scrambled shRNA showed major impairment in cell proliferation in various cell lines (data not shown), cells without doxycycline treatment were used as controls. Cells were exposed to thapsigargin up to 3 h and ER stress response was monitored by phosphorylation of eIF2α and PERK by western blotting. PDI KD almost completely abrogated eIF2α phosphorylation in HCT116 and HEK293T cells, indicating impaired PERK activity, although phosphorylation of PERK itself was still present (Fig. 1A, S1C). To ensure this phenotype was not only related to a disruption in calcium homeostasis, a second ER stressor tunicamycin, an inhibitor of N-linked glycosylation, was used in A549 and HEK293T PDI KD cells up to 5 h. In line with thapsigargin treatment, depletion of PDI resulted in impaired PERK signaling as shown by decreased eIF2α phosphorylation and BiP induction (Fig. 1B). The amount of total PERK protein was reduced during longer exposure times to tunicamycin, suggesting impaired stability or decreased expression (Fig. 1B). Treatment with the reducing agent DTT showed similar results (Fig. 1C).

**Figure 2:**
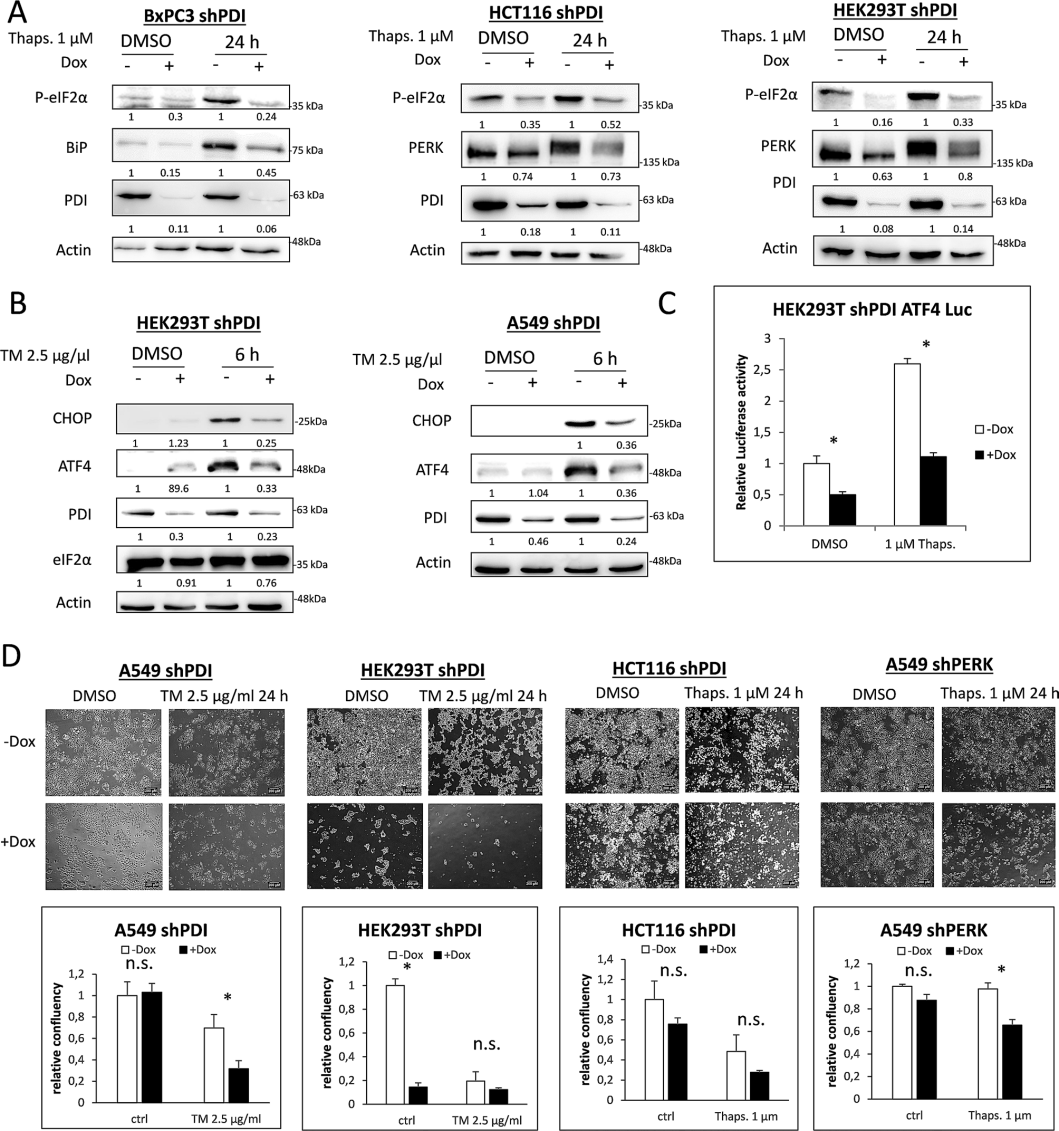
PDI is essential for PERK activation after long time exposure to ER stress. **(A)** KD of PDI was induced for 72 h in BxPC3, HCT116 and HEK293T cells. Cells were then treated with DMSO or 1 µM thapsigargin (Thaps.) for 24 h and phosphorylation of eIF2α, expression of BiP, PERK, PDI and Actin were monitored by western blotting. **(B)** HEK293T and A549 shPDI cells were treated with DMSO or 2.5 µg/ml tunicamycin (TM) for 6 h in the presence or absence of PDI. Expression of CHOP, ATF4, PDI, eIF2α and Actin were tested by western blot analysis. In subfigures A and B, densitometry was used to quantify protein band intensity. After normalization to Actin, the induced KD sample (+ Dox) was compared to its control sample (− Dox, set to 1). **(C)** ATF4 luciferase assay in HEK293T shPDI cells after 96 h of KD induction and 20 h of 1 µM thapsigargin treatment. **(D)** Bright-field microscopy pictures and quantification of cell confluence of A549, HEK293T and HCT116 shPDI cells after 96 h of KD induction and 24 h treatment with DMSO, 2.5 µg/ml tunicamycin (TM) or 1 µM thapsigargin (Thaps.). Magnification × 4, 2.5 NA.

ER stress is known to induce a pro-apoptotic signaling pathway through CHOP after long time exposure to ER stress inducing agents. To check if PERK activity was also decreased after PDI depletion in situations of long term exposure to ER stress, BxPC3, HCT116 and HEK293T cells were cultured in the presence of thapsigargin for 24 h. Depletion of PDI led to decreased eIF2α phosphorylation in all three cell lines, lower BiP induction in BxPC3 and declined levels of total PERK in HCT116 and HEK293T cells (Fig. 2A, S1C). Furthermore, CHOP and ATF4 were less induced without PDI in HEK293T and A549 cells after 6 h of tunicamycin treatment, indicating the necessity of PDI to induce full PERK downstream signaling (Fig. 2B). Luciferase assays were performed to validate transcriptional activity of ATF4 after PDI KD and 20 h of thapsigargin treatment in HEK293T cells. PDI KD significantly decreased ATF4 transcriptional activity already under basal conditions. Thapsigargin treatment did not lead to an adequate increase of ATF4 activity when PDI was depleted compared to the control cells, confirming the western blots results (Fig. 2C, S1A).

PERK activation has been shown to increase the survival of cells during ER stress by limiting protein translation and upregulation of important UPR target genes. Depletion of PDI combined with tunicamycin or thapsigargin treatment resulted in massive decrease of cell survival in A549, HEK293T and HCT116 cells as shown by bright-field microscopy, indicating higher vulnerability to ER stress which is in line with abrogation of the PERK pathway (Fig. 2D). KD of PERK in A549 cells showed comparable results (Fig. 2D).

### During the UPR, PDI specifically stabilizes PERK and does not promote IRE1 or ATF6 signaling

**Figure 3:**
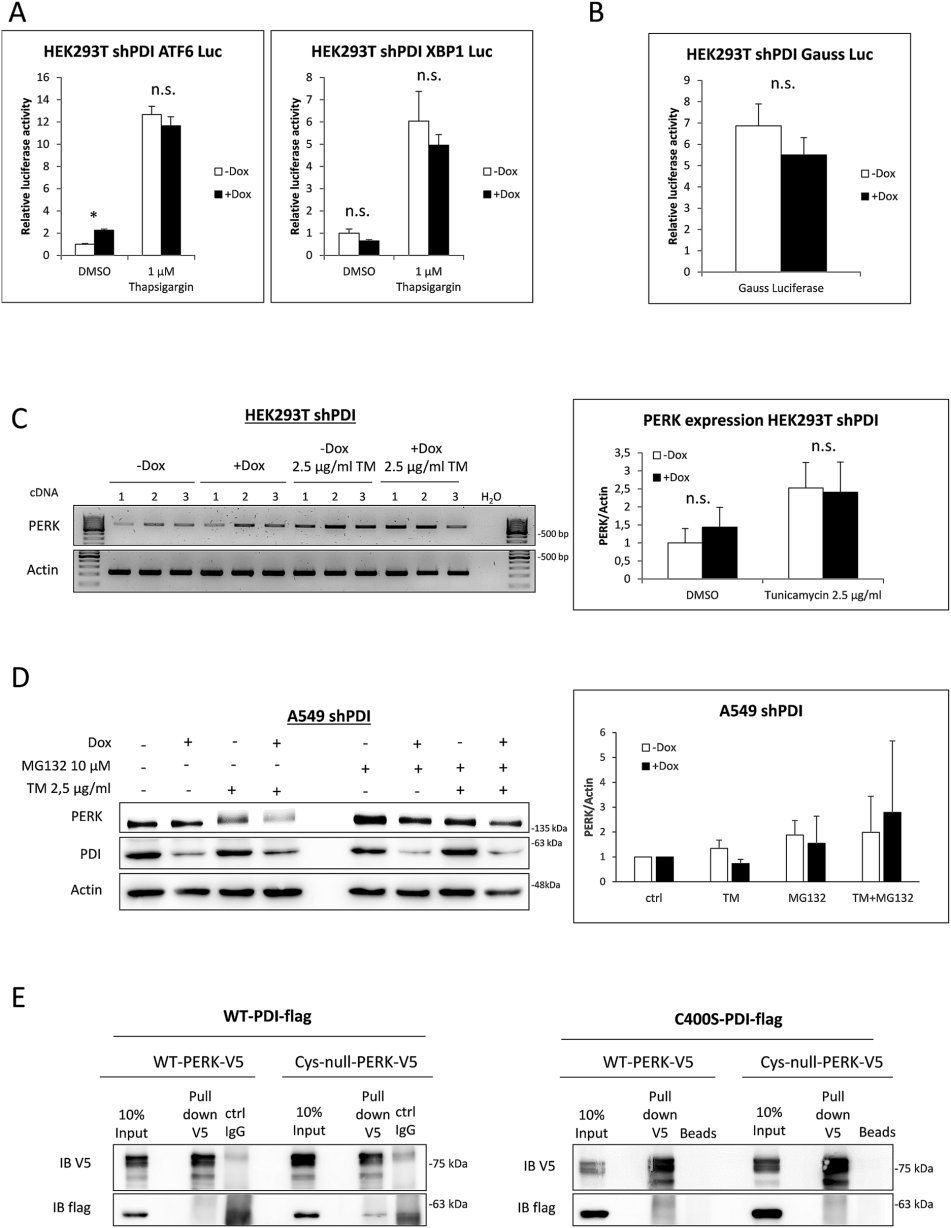
PERK is stabilized by PDI. **(A)** ATF6 and XBP1 luciferase assay in HEK293T shPDI cells. Cells were transfected with ATF6 and XBP1 reporter plasmids and treated with 1 µM thapsigargin for 20 h. **(B)** HEK293T shPDI cells were transfected Gauss luciferase and renilla luciferase plasmids. Activity of Gauss luciferase was measured in the supernatant and normalized to intracellular renilla luciferase after 72 h of KD induction. **(C)** RT-PCR of HEK293T shPDI cells for PERK and Actin using 3 independent cDNAs. Densitometry was used for quantification and is presented in bar graphs. **(D)** A549 shPDI cells were treated with 10 µM MG132 and 2.5 µg/ml tunicamycin for 4 h. Protein expression of PERK, PDI and Actin was monitored by western blotting. PERK protein levels were quantified using densitometry and the mean of three independent experiments are presented in bar graphs. **(E)** Co-immunoprecipitation experiments in HEK293T cells overexpressing PDI-WT-flag or C400S-PDI-flag together with the V5 tagged WT luminal domain or V5 tagged Cys-null luminal domain of PERK.

Luciferase assays were performed to assess if KD of PDI also affects IRE1 and ATF6 signaling (Fig. 3A). Under non-stressed conditions, we observed a minor increase in ATF6 activity and a slight decrease of XBP1 activity after KD of PDI. It became evident that KD of PDI alone does not induce full UPR signaling. However, under ER stress triggered by thapsigargin, ATF6 and IRE1 activity remained mainly unchanged through KD of PDI, indicating an exceptional role for PDI in activating the PERK pathway (compare Figs. 3A and 2C).

PDI is one of the most abundant proteins in the ER and is thought to contribute to disulfide bond formation of client proteins. To exclude that an overload of these client proteins without properly formed disulfide bonds is the cause for the increased vulnerability to ER stress, the activity of secreted gauss luciferase was monitored in the supernatant of PDI KD cells. Gauss luciferase contains five mandatory disulfide bonds to maintain luciferase function and therefore represents a good model for measuring protein folding and secretion ability of the ER. Notably, depletion of PDI did not significantly alter the activity of Gauss luciferase indicating that neither disulfide bond formation nor the secretory capacity was compromised (Fig. 3B).

PERK protein levels were decreased in PDI KD cells after short as well as long time exposure to ER stress inducing agents, indicating decreased PERK expression or increased PERK degradation when PDI is depleted (Figs. 1A-C and 2A,B). RT-PCR was performed to investigate differences in expression levels of PERK mRNA with and without tunicamycin treatment. However, no significant differences in transcriptional levels of PERK could be observed, strongly indicating a translational or posttranslational event (Fig. 3C). Since ER membrane proteins are usually degraded by the 26S proteasome in a p97 dependent manner ^31^, proteasomal degradation was blocked by MG132 prior to tunicamycin treatment and PERK protein levels were determined by western blotting. Indeed, MG132 treatment partly restored PERK protein levels after PDI KD in A549 cells, indicating a stabilizing function of PDI for PERK after its activation (Fig. 3D). Stabilization of PERK presumes a direct interaction between PDI and PERK which can occur by PDIs active cysteines at the a and a’ domain or its hydrophobic binding motif in the b’ domain. Co-immunoprecipitation experiments were performed in HEK293T cells expressing either the luminal domain of WT-PERK or Cys-null-PERK (4 luminal cysteines of PERK mutated to serine) together with WT-PDI or the trap mutant form C400S-PDI. These experiments showed no strong interaction between PERK and PDI, irrespective of the cysteines of PERK or expression of the PDI trap mutant which has been shown to enhance co-immunoprecipitation of PDI substrates (Fig. 3E). Anyhow, these results don’t exclude a possible transient interaction between PDI and PERK through the b’ motif during ER stress or an unknown necessary protein important for PERK activation which relies on functional PDI.

### Role of PDI in radiation sensitivity

**Figure 4:**
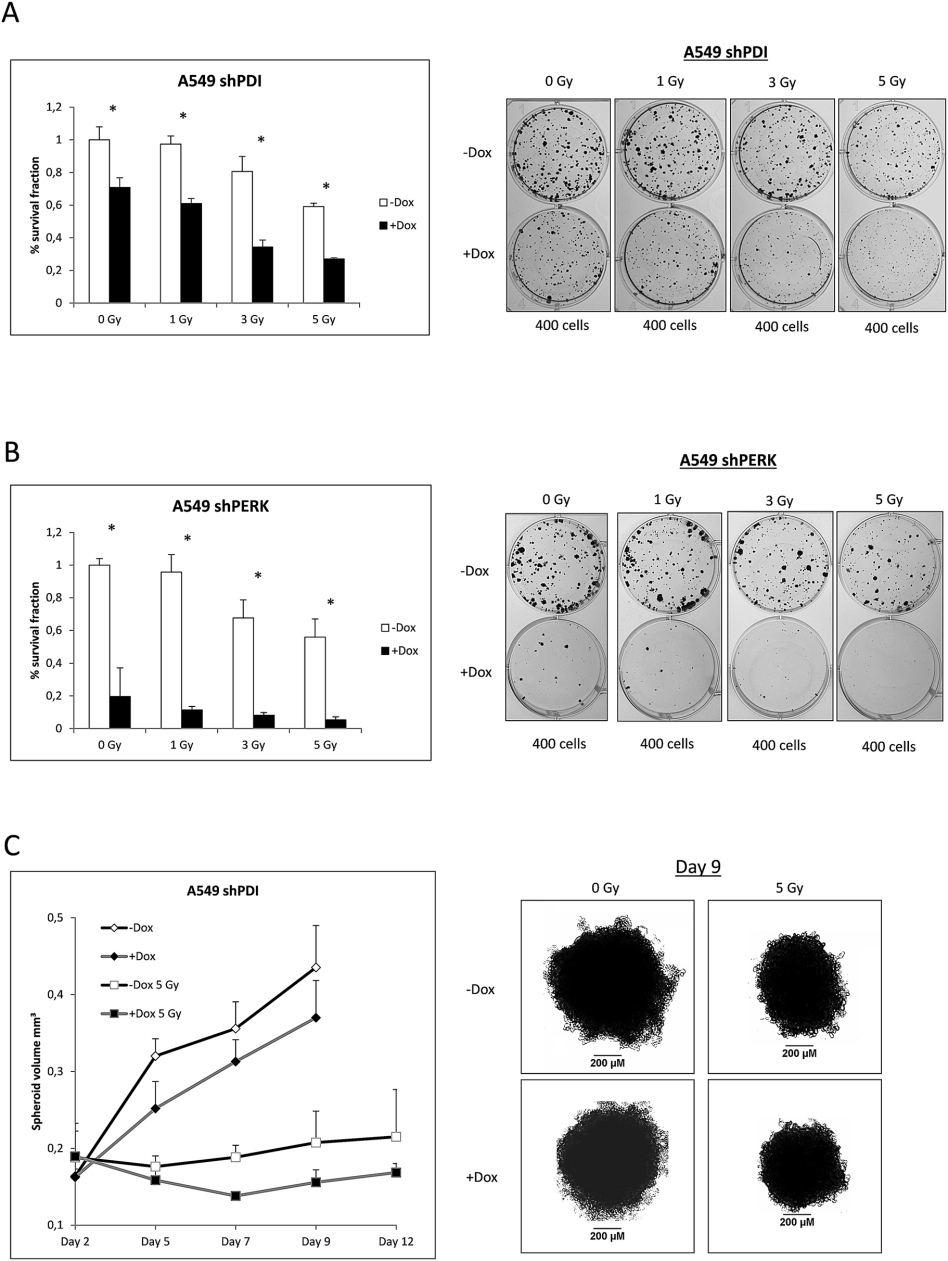
PDI and PERK promote survival after ionizing radiation. **(A,B)** Colony formation assay of A549 shPDI and shPERK cells irradiated with 0, 1, 3 and 5 Gy. Survival fraction is presented in bar graphs and representative images of colonies formed in 6-Well plates are shown. **(C)** Tumor spheroid growth experiment with A549 shPDI cells. Spheroids were irradiated with 5 Gy on day 2 and volume was measured on days 2, 5, 7, 9 and 12, n = 7. Representative images of tumor spheroids at day 9 are shown.

Radiotherapy has been shown to induce ER stress and activation of the PERK pathway. To elucidate the role of PDI and its activating function for PERK during radiotherapy, colony formation assays were performed. KD of PDI decreased colony formation ability of A549 cells already under control conditions and was further reduced after different doses of radiation (Fig. 4A, S1D). The same tendencies were observed using an shRNA against PERK, although the effect was even more pronounced (Fig. 4B). To simulate in vivo tumor conditions, spheroid growth experiments were carried out. KD of PDI combined with radiation decreased the spheroid volume during the first days after radiation and resulted in later regrowth compared to the control spheroids (Fig. 4C). These results highlight the mandatory protective function of PDI and PERK under ER stress conditions and strongly suggest PDI as a target to reduce adaptation to ER stress through the PERK pathway.

## Discussion

The most common theory how ATF6, IRE1 and PERK are activated in response to ER stress to date is the dissociation of BiP, which then allows dimerization and autophosphorylation for IRE1 and PERK or cleavage of ATF6 respectively. Nevertheless, there is still uncertainty about the binding of BiP, its dissociation, ATP dependence, mandatory cofactors and redox regulation of luminal cysteines regarding to the activation and fine tuning of these sensors^7,32^.

In this study we were able to almost completely abrogate PERK signaling by depletion of PDI in HEK293T, BxPC3, A549 and HCT116 cells. This phenotype was explained by decreased stability of activated PERK and increased degradation of the UPR sensor by the 26S proteasome after loss of PDI. Expression level of PERK mRNA remained unchanged and the induction during ER stress appeared to be similar after depletion of PDI. It is possible that due to missing eIF2α phosphorylation the cap independent translation of PERK mRNA is not as effective after PDI KD as compared to control cells. This could also contribute to the decreased PERK protein level we observed after long time exposure to chemical ER stress inducers.

PERK possesses 4 luminal cysteines, but their roles in activation, inactivation or stability of PERK remain elusive. Although we could not show a direct interaction between PERK and PDI, it is not unlikely that PDI contributes directly or indirectly to proper disulfide arrangement in the luminal domain of PERK which in turn prevents degradation. The significance of luminal cysteines has been already shown for IRE1 and ATF6. Eletto et al. could prove that PDIA6 limits the duration of IRE1 signaling by direct binding to cysteine 148 to adjust IRE1 activity and thereby preventing apoptosis^8^. Furthermore, they showed a direct binding between PERK and PDIA6 regulating the strength and duration of PERK activity, which could be the missing link between PDI and PERK in our observed phenotype^8^. ATF6 exists in monomers as well as disulfide bonded dimers and oligomers in unstressed cells^11^. The level of intramolecular disulfide reduction correlates with ATF6 transcriptional activity and is therefore a mandatory event to activate this sensor^11^. Additionally, PDIA5 has been identified to be mandatory for this disulfide bond rearrangement during ER stress, leading to ATF6 export from the ER and enhanced chemoresistance^10^. A recent study identified CNPY2 as a key factor for activating and maintaining PERK function. CNPY2 is released from BiP during ER stress and engages PERK signaling by direct binding in mice and mice cells^12^. CNPY2 exists in disulfide bonded dimers and therefore could be a PDI client protein, although the binding to PERK seemed to be independent of CNPY2 cysteines^12^. However, we did not see a decrease in CNPY2 expression or dimer formation after depletion of PDI (data not shown). In our hands, BiP levels were decreased in PDI KD cells during ER stress and therefore CNPY2 should be able to bind PERK to a higher extend and enhance PERK activity even more, which was clearly not the case. Furthermore, we were unable to detect CNPY2 expression in A549 cells and therefore excluded it as the key factor for our observed results.

**Figure 5:**
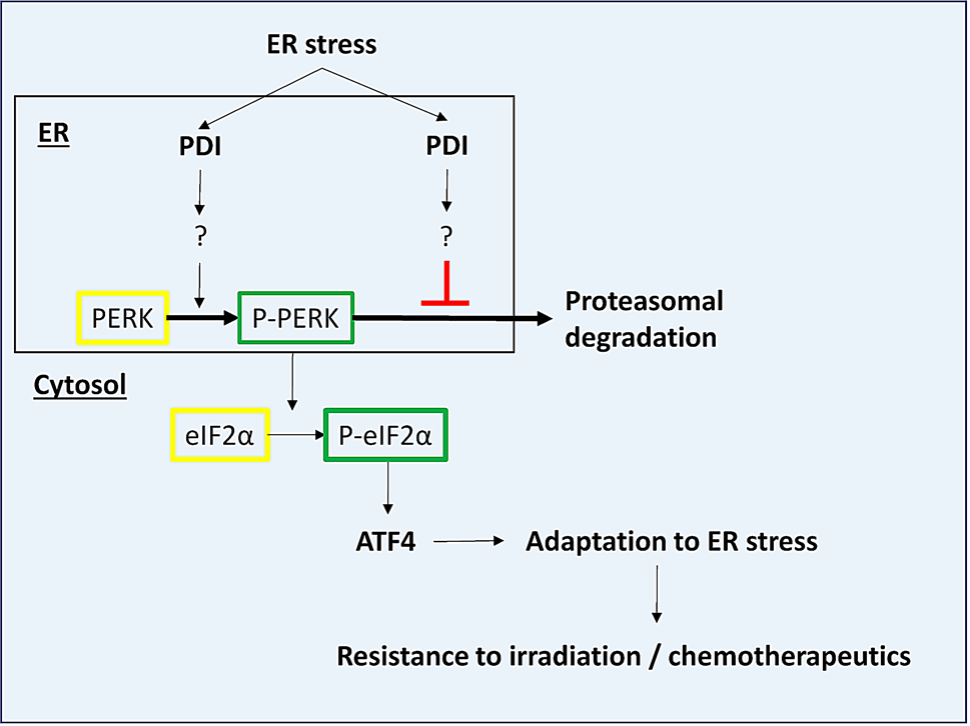
Model of PDI’s dual role for PERK pathway activation under ER stress in cancer cells.

Based on our results we suggest a dual role of PDI for the PERK pathway which is depicted in Fig. 5. Herein, PDI is not only indispensable for PERK activation but also prevents its degradation. Although the precise mechanism how PDI is involved in PERK signaling remains uncertain, this observation shows potential for clinical applications. Activation of the UPR and especially the PERK pathway are present in almost all tumors and support tumor growth and adaptation to nutrient deprivation and hypoxia^13,15,17,20,33^. It is noteworthy that loss of PERK can be compensated by GCN2 as well as GCN2 loss can be compensated by PERK in some tumor models, which in part negotiates the antitumor effects^34,35^. We could not observe any compensation after loss of PDI regarding to ATF4 induction and further PERK downstream signaling. This might be explained through a lesser extend of PERK blockade which not induces compensation by GCN2, maybe due to incomplete PDI KD or by also affecting GCN2 kinase activity. Furthermore, the defect in PERK activity was not accompanied by increased activation of ATF6 or IRE1.

Anyhow, depletion of PDI increased the sensitivity of all tested cells to ER stress inducing agents and ionizing radiation with comparable effects like the direct KD of PERK. This was further validated using a tumor spheroid model, which simulates the tumor growth and radiation response in vivo more precise^36^. While PERK inhibition or depletion in tumor cells remarkably reduced tumor growth and metastasis in mouse models^21^, clinical applications of PERK inhibitors are limited by pancreatic adverse effects^23,37^. Different PDI inhibitors in comparison were well tolerated in preclinical tumor models and showed strong antitumor activity^38–42^. The mechanisms of action of PDI inhibitors vary massively regarding to the effect on UPR pathways. While in some studies UPR activation is not addressed^42^, others show massive induction of all UPR pathways in multiple myeloma cells^41^ or no effect on the UPR at all^38^. It is worth noting, that although most inhibitors targeting the enzymatic activity of PDI at the a domain (PACMA31, KSC-34, CCF642), differences in chemical structure seem to alter UPR response after inhibition and therefore off target effects cannot be excluded. To our knowledge, studies comparing genetic depletion of PDI with these inhibitors have not been executed yet. It is therefore difficult to distinguish which roles the a’ and the b domains play after inhibition of the a domain and if their functions are also affected. Another PDI modulator, LOC14, which forces PDI to adapt in an oxidized conformation and thereby blocks enzymatic activity, has been reported to suppress UPR response in a mutant huntington model and supports our findings that PDI is important for activating the PERK pathway^43,44^. To address these indistinct results, further research using genetic knockout models and re-expressing mutant forms of PDI with dysfunctional enzymatic activity are necessary to delineate the functions of the different PDI domains regarding their involvement in sensing ER stress.

In summary, PDI presents a novel target to selectively overcome tumor cell resistance to ER stress and radiotherapy acquired by activation of the PERK pathway in solid as well as leukemic malignancies while sparing the severe side effects received by direct inhibition of PERK.

## Material and methods

### Cell culture, transfection and lentiviral transduction

HEK293T (ATCC, CRL-3216, human embryonic kidney) and A549 (ATCC, CCL-185 human lung adenocarcinoma) cells were cultured in DMEM high glucose (Invitrogen, Darmstadt, Germany), BxPC3 (DSMZ, ACC-760, human pancreatic adenocarcinoma) cells in RPMI 1640 (Invitrogen) and HCT116 (ATCC, CCL-247, colorectal carcinoma) cells in McCoy’s 5A (Pan-Biotech, Aidenbach, Germany) supplemented each with 10% tetracycline-free FBS and antibiotics. Transient transfections were performed using Viafect (Promega, Mannheim, Germany). Production of lentiviral particles were done in HEK293T cells as described previously (Kranz et al., 2017). For transduction, 2 × 10^5^ cells were incubated with 2 × 10^6^ lentiviral particles in the presence of 8 µg/ml polybrene for 24 h followed by selection with 2 µg/ml puromycin for 5 days. All experiments were performed with mycoplasma-free cells.

### Plasmids, shRNA sequences and knockdown induction

Plasmids used for luciferase assays: pFLAG-XBP1u-FLuc (Addgene #31239), p5xATF6-GL3 (#11976, Addgene), pNL NlucP/ATF4-RE/Hygro (Promega), pTK-GLuc- (#N8084S, NEB, Frankfurt/Main, Germany), pGL4.74 (#E6921, Promega). pCMMP-dnPERK-IRES-eGFP (Addgene #36954) was used to create V5-tagged and Cys-null mutant (C187S, C192S, C307S, C423S) of PERK luminal domain by site directed mutagenesis^45^. The following shRNA sequences were cloned into Tet-pLKO vector: 5′-GTGTGGTCACTGCAAACAGTT-3′ (corresponding to 1,385–1,405 bp of human PDI mRNA, GenBank acc. no. NM_000918), #2 5′-TGCTGTTCTTGCCCAAGAGTG-3′ (corresponding to 837–857 bp of human PDI mRNA, GenBank acc. no. NM_000918), 5′-GGAACGACCTGAAGCTATAAA-3′ (corresponding to 3,383–3,403 bp of human PERK mRNA, GenBank acc. no. NM_001313915.1). Knockdown of PDI and PERK was induced by addition of 250 ng/ml doxycycline to the cell culture media.

### RNA extraction, RT-primer and PCR

RNA extraction was performed with the NEB monarch RNA extraction kit (NEB) following the manufacturer’s instructions. cDNA was generated using the QuantiTect Reverse Transcription Kit (Qiagen, Hilden, Germany). For RT-PCR SampleIN Direct PCR Kit (highQu, Kraichtal, Germany) was used according to the manufacturer’s protocol. Expression of PERK was tested by using PERK primers 5′-ATCCCCCATGGAACGACCTG-3′ (forward), 5′-ACCCGCCAGGGACAAAAATG-3′ (reverse). β-Actin expression was assessed using Actin primers 5′-GCCGCCAGCTCACCAT-3′ (forward) and 5′-TCGATGGGGTACTTCAGGGT-3′ (reverse). Conditions for amplifications: 95 °C for 1 min followed by 30 cycles of 95 °C for 30 s, 58 °C for 30 s and 72 °C for 30 s. Samples were loaded on a 2% agarose gel containing SYBR Safe DNA Gel stain (Thermo fisher scientific, Schwerte, Germany). Bands were visualized using the FX7 chemoluminescence documentation system (Peqlab, Erlangen, Germany) and quantified using ImageJ software.

### Antibodies and reagents

Tunicamycin, Thapsigargin and MG132 were obtained in p.a. quality from Sigma-Aldrich (Schnelldorf, Germany) and Santa Cruz biotechnology (Santa Cruz, California, US). Antibodies against GAPDH and Actin were from Sigma-Aldrich, against PDI from RnD-Systems (Minneapolis, MN, USA), against BiP, eIF2α, P-eIF2α, PERK, PDI, CHOP, V5, Flag and ATF4 from Cell-Signaling technologies (Frankfurt/Main, Germany). Secondary HRP coupled antibodies against rabbit and mouse were from DAKO (Hamburg, Germany).

### Clonogenic survival assay

400 A549 cells were plated in six-well plates and KD was induced immediately afterwards. Cells were irradiated with different doses after 40 h using an X-ray machine (X-rad 320, PXI) operated at 320 kV, 12.5 mA with a 1.65 mm aluminum filter (dose rate: 3.6 Gy/min). After 11 to 13 days of incubation, colonies were fixed and stained with 0.25% PFA, 70% EtOH and Coomassie brilliant blue (0.1 Coomassie blue, 5% acetic acid, 45% methanol). Colonies were counted automatically by using the colony area plugin for ImageJ as described previously^46^. Plating efficiency (PE) and survival fraction (SF) were calculated with the formulas “PE = colonies formed/number of cells seeded” and “SF = colonies formed/number of cells seeded × PE”.

### Tumor spheroid growth experiments

Tumor spheroid growth was performed in 96-well round bottom plates coated with 1.5% agarose. 5 × 10^3^ A549 shPDI cells were seeded, KD was induced immediately and plates were centrifuged with 800 rpm for 30 min. After 2 days, spheroids were measured and irradiated with 5 Gy. Spheroid area was measured using ImageJ plugin as described previously ^47^ and volume was calculated using the sphere formula (4/3 * π * r^3^). Controls had to be terminated before treated samples due to exponential growth and rapidly exceeding the size of the well.

### Cell lysis, SDS-PAGE and western blotting

Cells were seeded in 6-Well or P60-dishes at densities between 7 × 10^4^ and 2 × 10^5^ cells per well and KD was induced directly afterwards. After 72 h cells were treated with ER-stress inducers for the indicated time and lysed in RIPA buffer (50 mM Tris pH 7.5, 2 mM EDTA, 150 mM NaCl, 1% Nonidet P40, 0.1% SDS, 0.5% sodium deoxycholate and protease/phosphatase inhibitor cocktail (Cell Signaling)). Protein concentration was measured with a BCA assay kit (Thermo fisher scientific) and 10–30 µg protein were dissolved in SDS sample buffer (62.5 mM Tris (pH 7.4)) 2% SDS, 3% β-mercaptoethanol, 10% glycerol, 0.25 mg/ml bromophenol blue, 25 mM DTT). Samples were loaded on 7.5–10% polyacrylamide gels and afterwards transferred to PVDF membranes using the Trans-Blot Turbo system (Bio Rad, Munich, Germany). Membranes were blocked in 5% skim milk in TBS-T (50 mM Tris/HCL, 150 mM, NaCl, 0.1% Tween-20, pH 7.5). Primary antibodies were diluted as recommended by the manufacturer and incubated with the membrane overnight. Secondary antibodies were diluted 1:2000 in TBS-T and incubated for 1 h. ECL kit (Thermo Fisher Scientific, Oberhausen, Germany) and FX7 chemiluminescence documentation system (Peqlab) were used for protein detection. To show results of the western blots thoroughly through the whole manuscript, we avoided presenting multiple gels in one subfigure. Therefore, it was not possible to present all targets of interest in every subfigure.

### Co-immunoprecipitation

8 × 10^4^ HEK293T cells were plated in four wells of a 24-well plate and transfected with 500 ng V5-PERK and 500 ng flag-PDI per well. After 72 h of incubation four wells were pooled together to achieve adequate protein concentration for immunoprecipitation. Cells were lysed in caspase lysis buffer (Tris (pH 7.3) 50 mM, NaCl 150 mM, NP-40 1% (v/v)) and incubated for 4 h at 4 °C with V5 antibody. Afterwards the lysate was incubated for 1 h with protein S and G magnetic beads (Cell-Signaling) for pull down. Lysates were transferred to SDS-sample buffer containing DTT and heated at 95 °C for 10 min before loaded on a 7.5% polyacrylamide gel.

### Luciferase assays

7 × 10^4^ HEK293T cells were seeded in 24-well plates, KD was induced immediately and cells were transfected with 500 ng of firefly luciferase reporter gene plasmid and 100 ng of renilla luciferase plasmid. After 72 h cells were harvested and prepared for measurement using the Dual Glo luciferase assay kit (Promega) as recommended by the manufacturer. Firefly and renilla luciferase were measured using the GloMax detection system (Promega) and normalized to renilla values to exclude variations in transfection efficiency. For testing the secretion efficiency, cells were transfected with 500 ng gaussia luciferase and 100 ng of renilla luciferase plasmid. Luciferase activity was measured in cell culture media and normalized to intracellular renilla luciferase activity.

### Microscopy

For bright field microscopy images a Zeiss Axiovert 200M (Carl Zeiss, Oberkochen, Germany) was used with a 4 × magnification objective and 2.5 NA. Quantification of cell confluence was performed using ImageJ.

### Statistical analysis

All results were obtained in at least two independent experiments. In bar graphs mean plus standard deviation is shown for one representative experiment run in triplicates. For comparison between groups, two-way ANOVA with Bonferroni or Sidak post-hoc test was used. Significance is presented as *P < 0.05.

## Supplementary information

Supplementary Figure S1.

Supplementary Information.

## Data Availability

The data that support the findings of this study are available from the corresponding author upon reasonable request.

## Abbreviations

ATF4: Activating transcription factor 4
ATF6: Activating transcription factor 6
BiP: Binding immunoglobulin protein
CHOP: C/EBP-homologous protein
CNPY2: Protein canopy homolog 2
DMSO: Dimethyl sulfoxide
Dox: Doxycycline
DTT: Dithiothreitol
eIF2α: Eukaryotic initiation factor 2
ER: Endoplasmic reticulum
ERp46: ER protein 46
ERp57: ER protein 57
GAPDH: Glyceraldehyde 3-phosphate dehydrogenase
GCN2: General control nonderepressible 2
IRE1: Inositol-requiring enzyme 1
KD: Knockdown
PDI: Protein disulfide isomerase
PERK: Protein kinase R (PKR)-like endoplasmic reticulum kinase
ROS: Reactive oxygen species
RT-PCR: Reverse transcription polymerase chain reaction
Thaps: Thapsigargin
TM: Tunicamycin
UPR: Unfolded protein response
WT: Wildtype
XBP1: X-box binding protein 1
