# Supplementary material for: Tumor cells rely on the thiol oxidoreductase PDI for PERK signaling in order to survive ER stress
Source: Sci Rep. 2020 Sep 17;10:15299. doi: 10.1038/s41598-020-72259-1 (PMC7499200; doi:10.1038/s41598-020-72259-1)
Supplement: Supplementary file 1 — Supplementary Figure S1. [file 41598_2020_72259_MOESM1_ESM.pdf]

Tumor cells rely on the thiol oxidoreductase PDI for PERK signaling in order to survive ER stress

Philip Kranz, Christopher Sänger, Alexandra Wolf, Jennifer Baumann, Eric Metzen, Melanie Baumann, Kirsten Göpelt, Ulf Brockmeier[1]

Institut für Physiologie, Universität Duisburg-Essen, Germany

[1] Corresponding author: Dr. Ulf Brockmeier, PhD, Department of Neurology, University Hospital Essen, University Duisburg-Essen, Germany, Email ulf.brockmeier@uni-due.de

Supplementary data

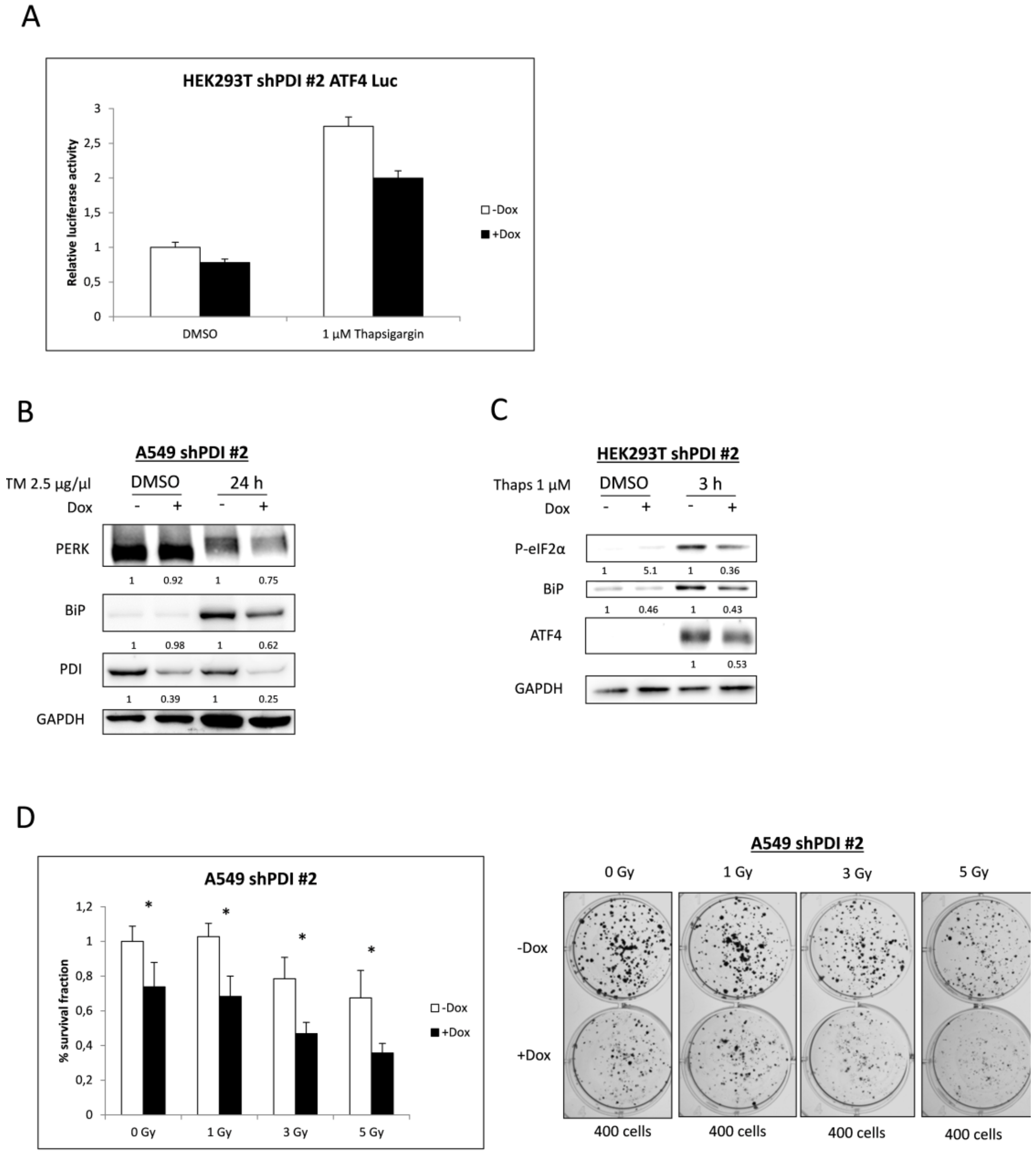

Figure S1: Key experiments with second shRNA (A) ATF4 luciferase assay in HEK293T shPDI #2 cells after 96 h of KD induction and 20 h treatment with 1 µM thapsigargin. **(B)** A549 shPDI #2 cells were treated with 2.5 µg/ml tunicamycin (TM) for 24 h after 48 h of KD induction and expression of PERK, BiP, PDI and GAPDH were tested by western blotting. **(C)** HEK293T shPDI #2 cells were treated with DMSO or 1 µM thapsigargin (Thaps.) for 3 h after 72 h of KD induction. Phosphorylation of eIF2α and expression of BiP, ATF4 and GAPDH were tested by western blotting. **(D)** Colony formation assay of A549 shPDI #2 cells irradiated with 0, 1, 3 and 5 Gy. Survival fraction is presented in bar graphs and representative images of colonies formed in 6-Well plates are shown. In subfigures B and C, densitometry was used to quantify protein band intensity. After *normalization to GAPDH,* the induced KD sample (+Dox) was compared to its control sample (-Dox, set to 1).
