# Supplementary material for: Tumor cells rely on the thiol oxidoreductase PDI for PERK signaling in order to survive ER stress
Source: Sci Rep. 2020 Sep 17;10:15299. doi: 10.1038/s41598-020-72259-1 (PMC7499200; doi:10.1038/s41598-020-72259-1)
Supplement: Supplementary file 2 — Supplementary Information. [file 41598_2020_72259_MOESM2_ESM.pptx]

## Slide 1
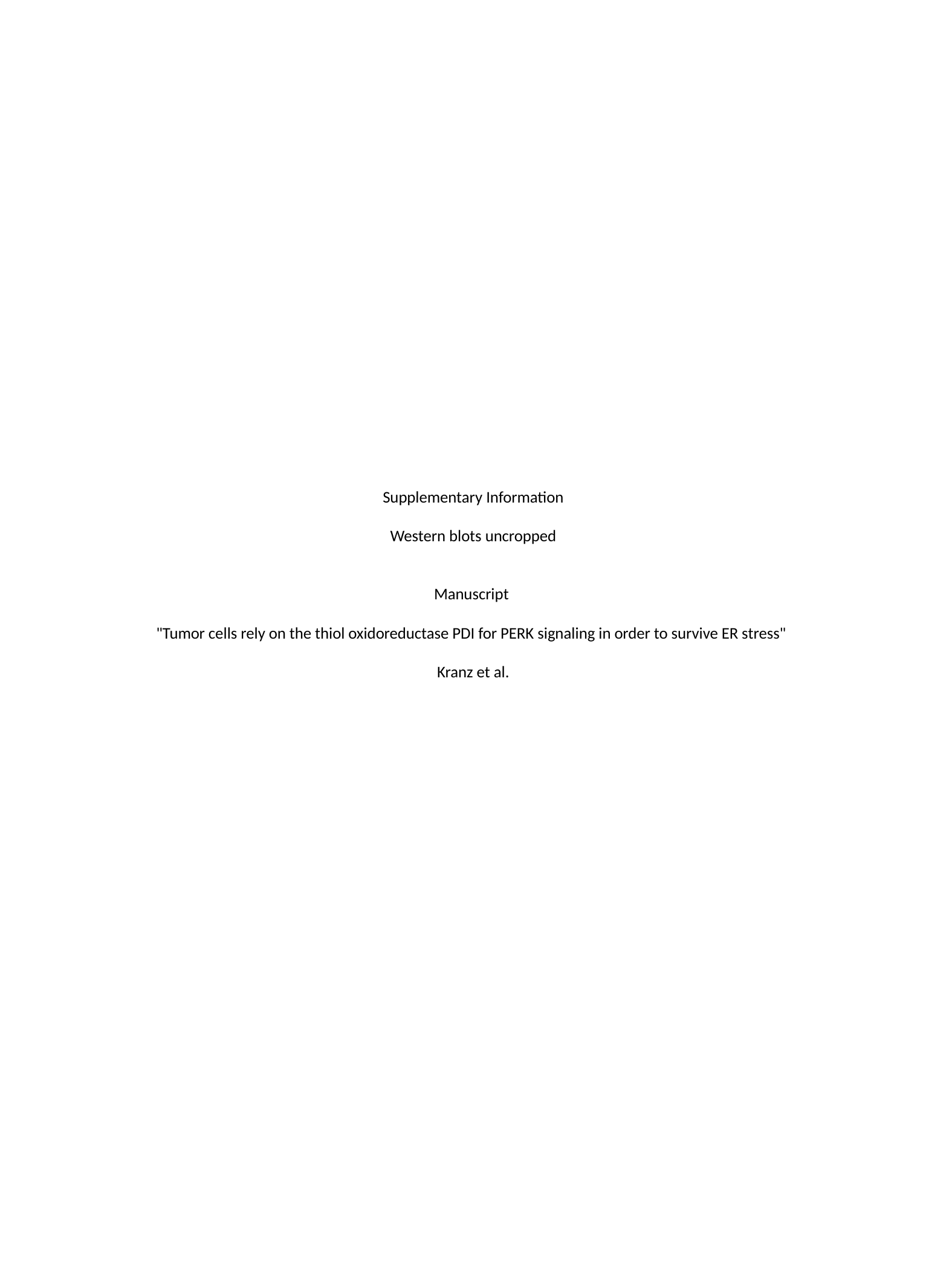

# Supplementary Information Western blots uncropped Manuscript "Tumor cells rely on the thiol oxidoreductase PDI for PERK signaling in order to survive ER stress"  Kranz et al.

## Slide 2
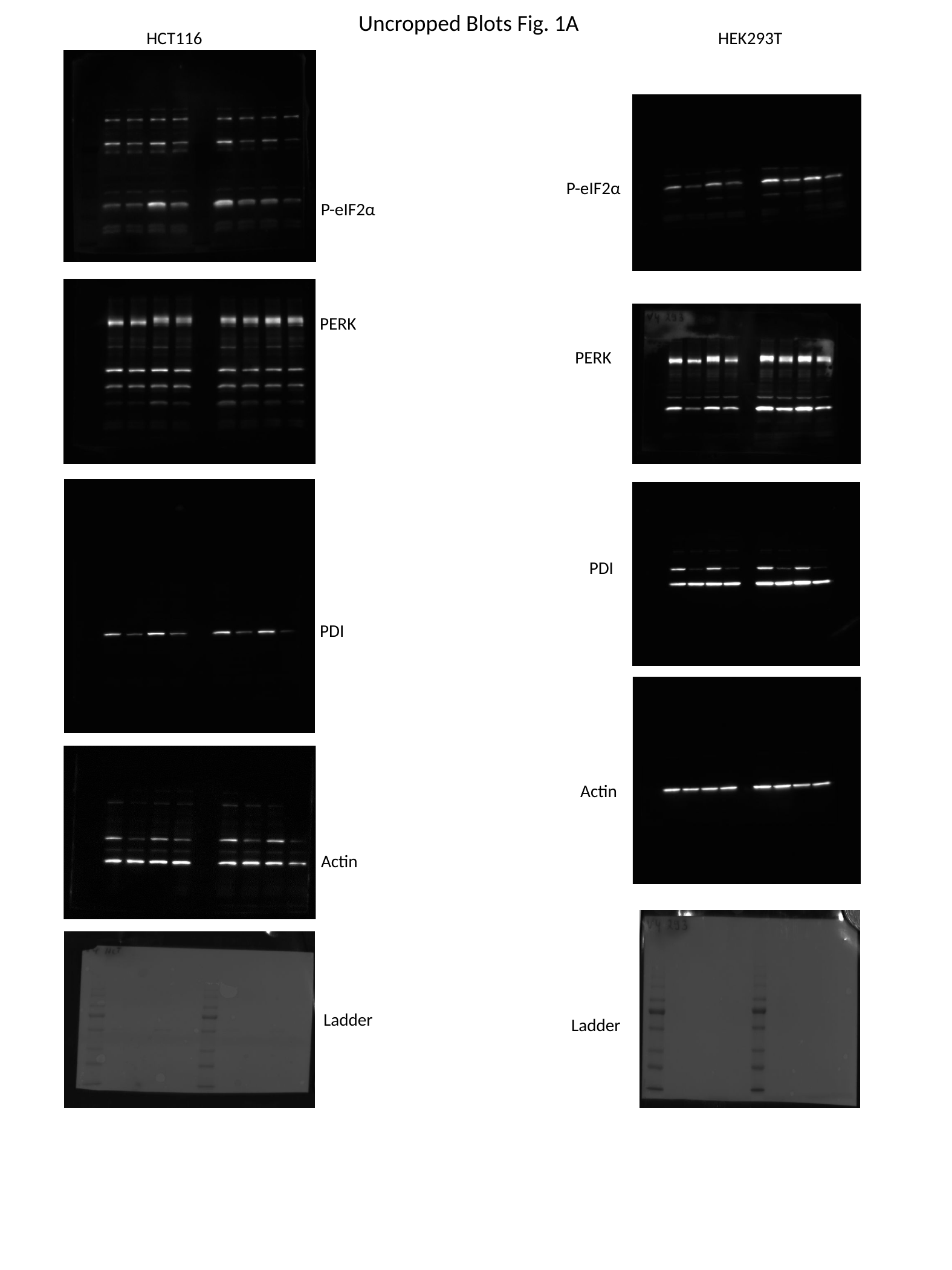

Uncropped Blots Fig. 1A
HCT116
HEK293T
P-eIF2α
P-eIF2α
PERK
PERK
PDI
PDI
Actin
Actin
Ladder
Ladder

## Slide 3
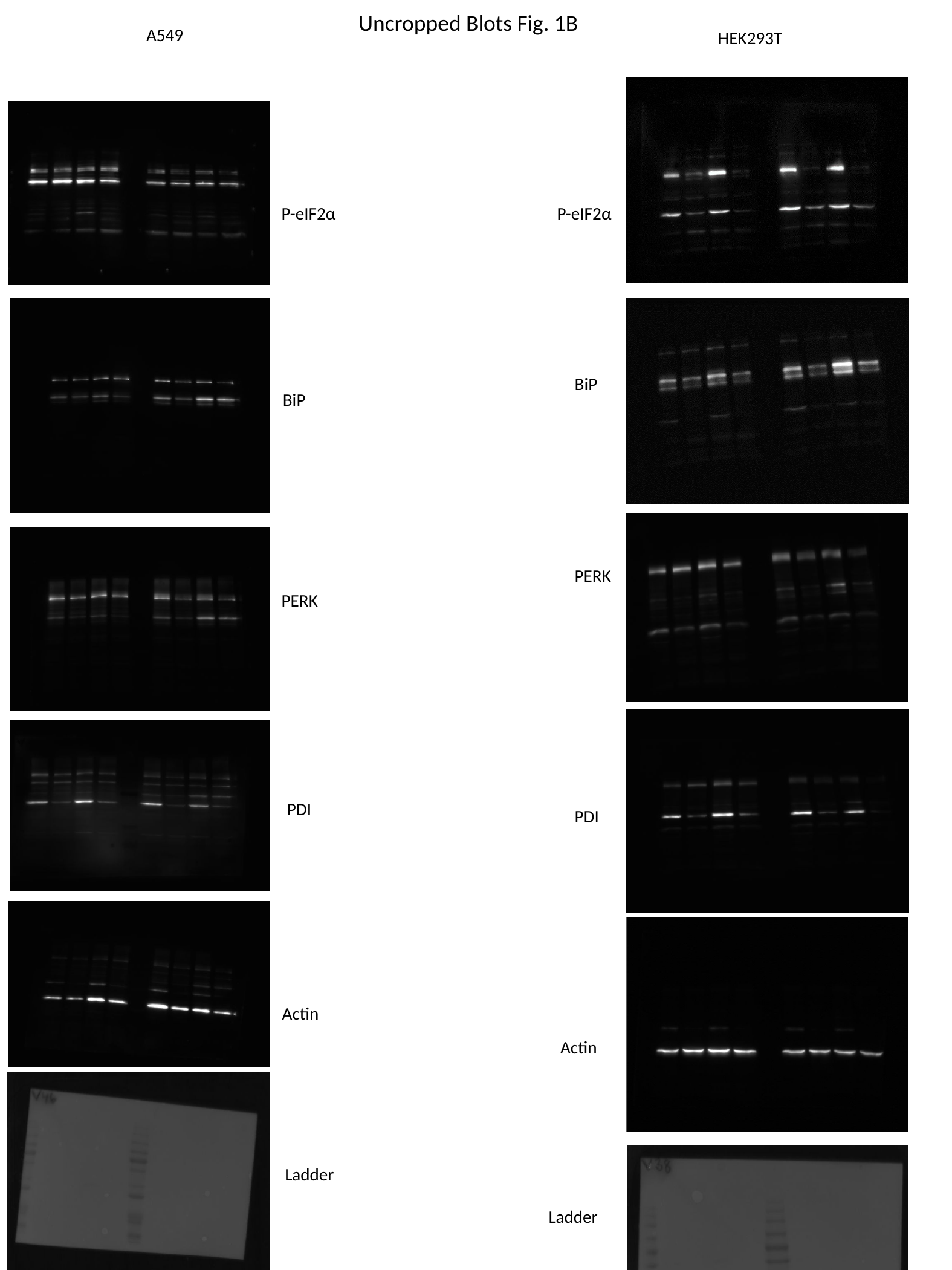

Uncropped Blots Fig. 1B
A549
HEK293T
P-eIF2α
P-eIF2α
BiP
BiP
PERK
PERK
PDI
PDI
Actin
Actin
Ladder
Ladder

## Slide 4
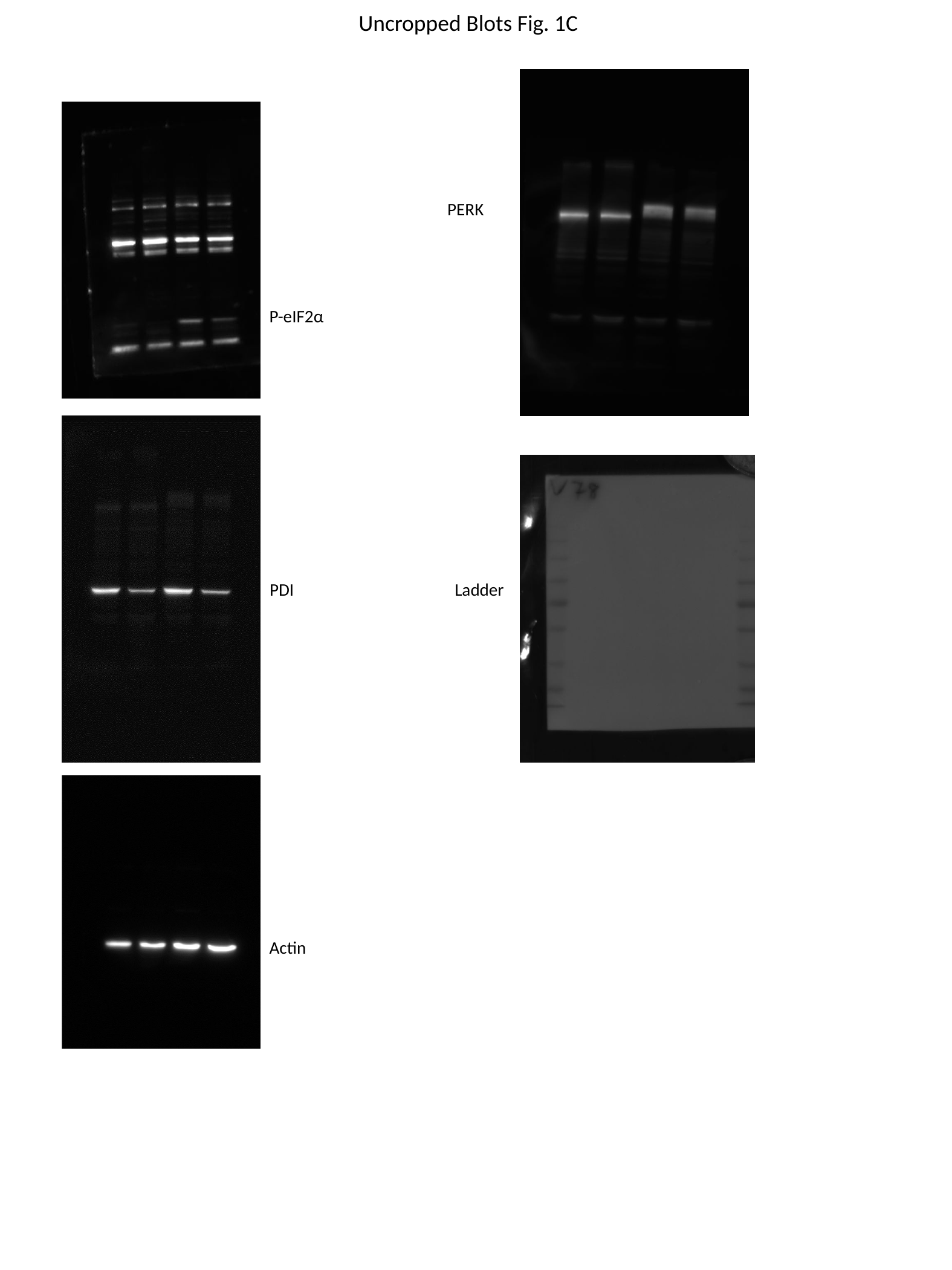

Uncropped Blots Fig. 1C
PERK
P-eIF2α
PDI
Ladder
Actin

## Slide 5
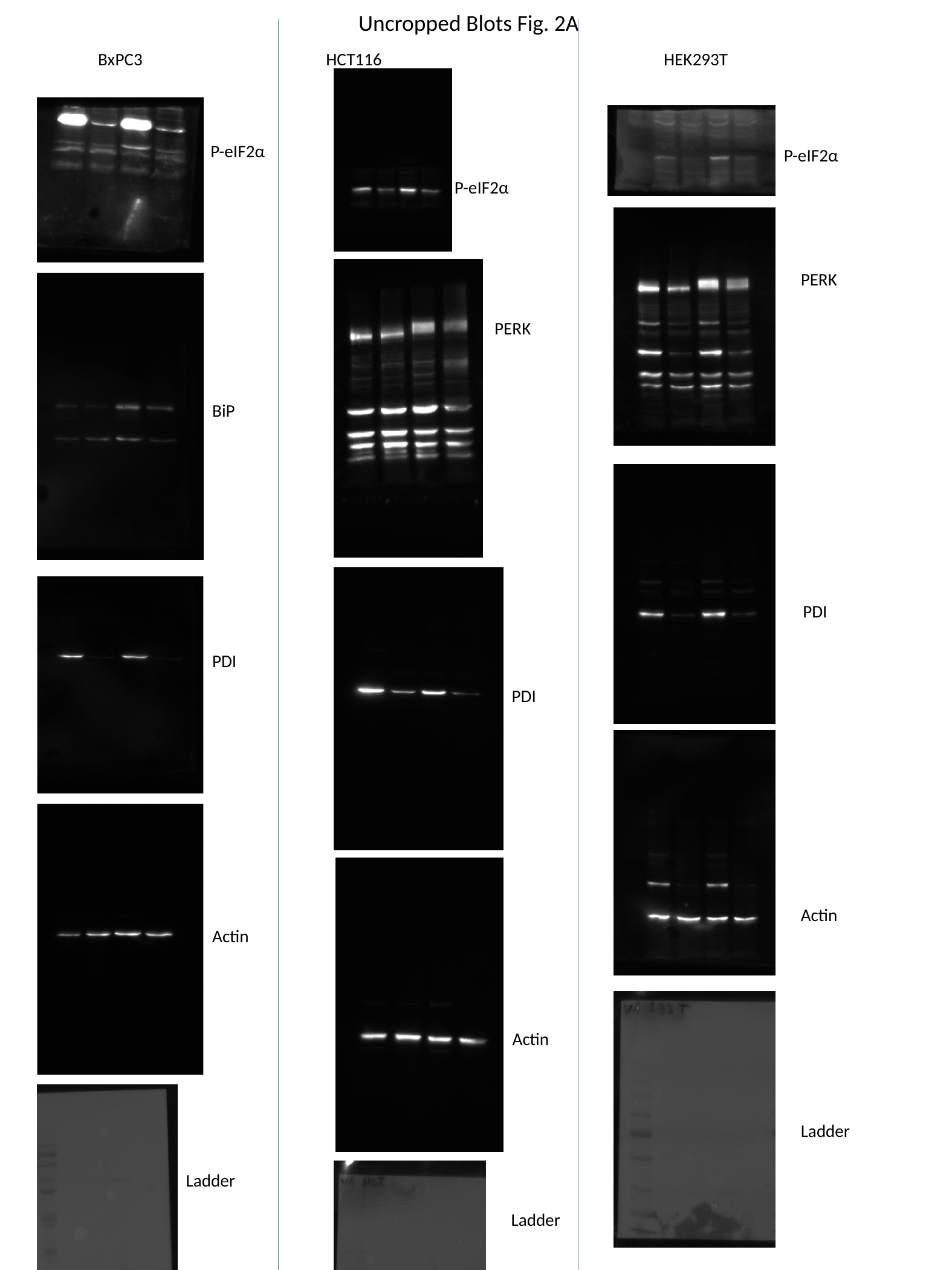

Uncropped Blots Fig. 2A
HEK293T
BxPC3
HCT116
P-eIF2α
P-eIF2α
P-eIF2α
PERK
PERK
BiP
PDI
PDI
PDI
Actin
Actin
Actin
Ladder
Ladder
Ladder

## Slide 6
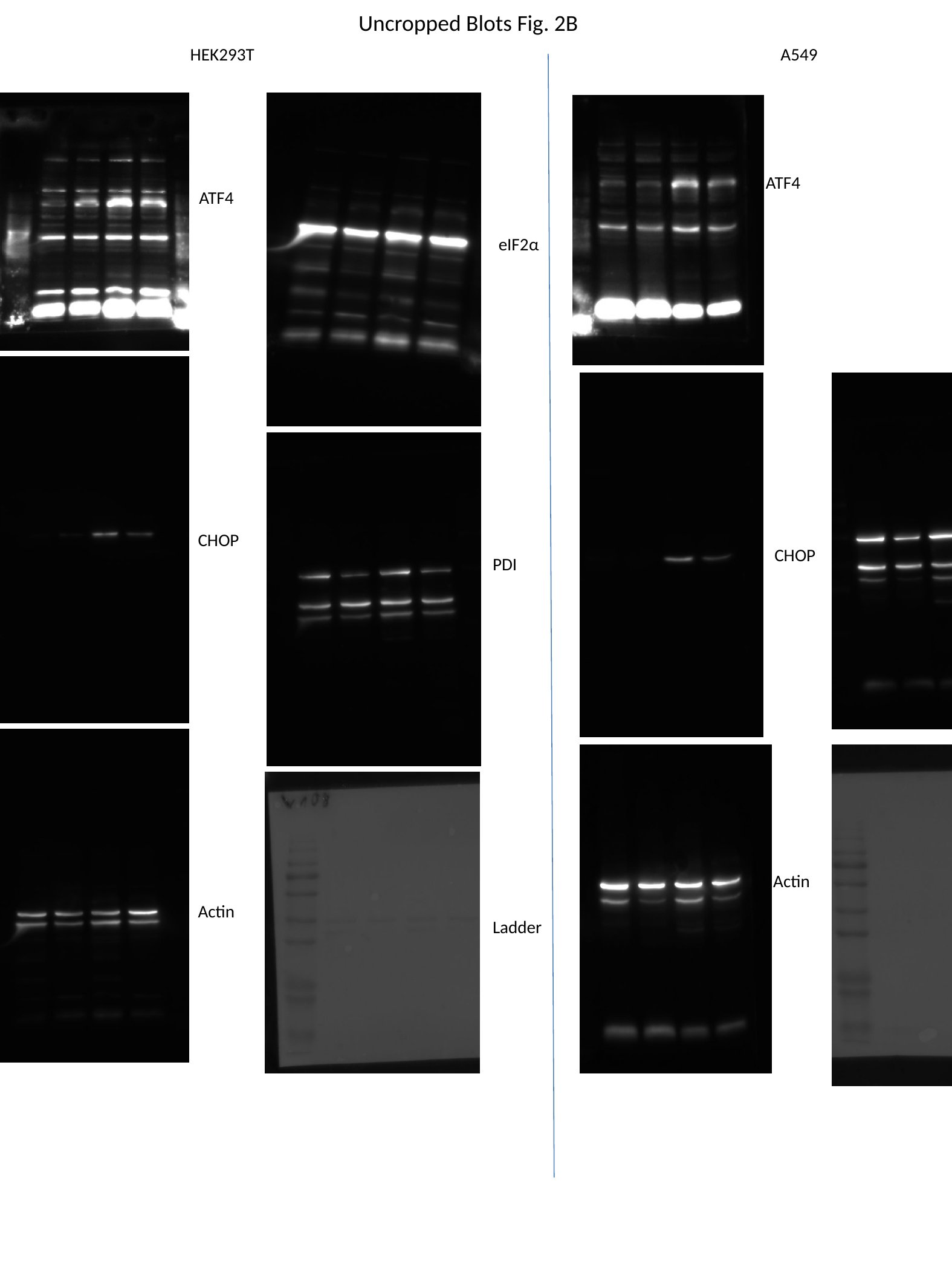

Uncropped Blots Fig. 2B
HEK293T
A549
ATF4
ATF4
eIF2α
PDI
CHOP
CHOP
PDI
Actin
Actin
Ladder
Ladder

## Slide 7
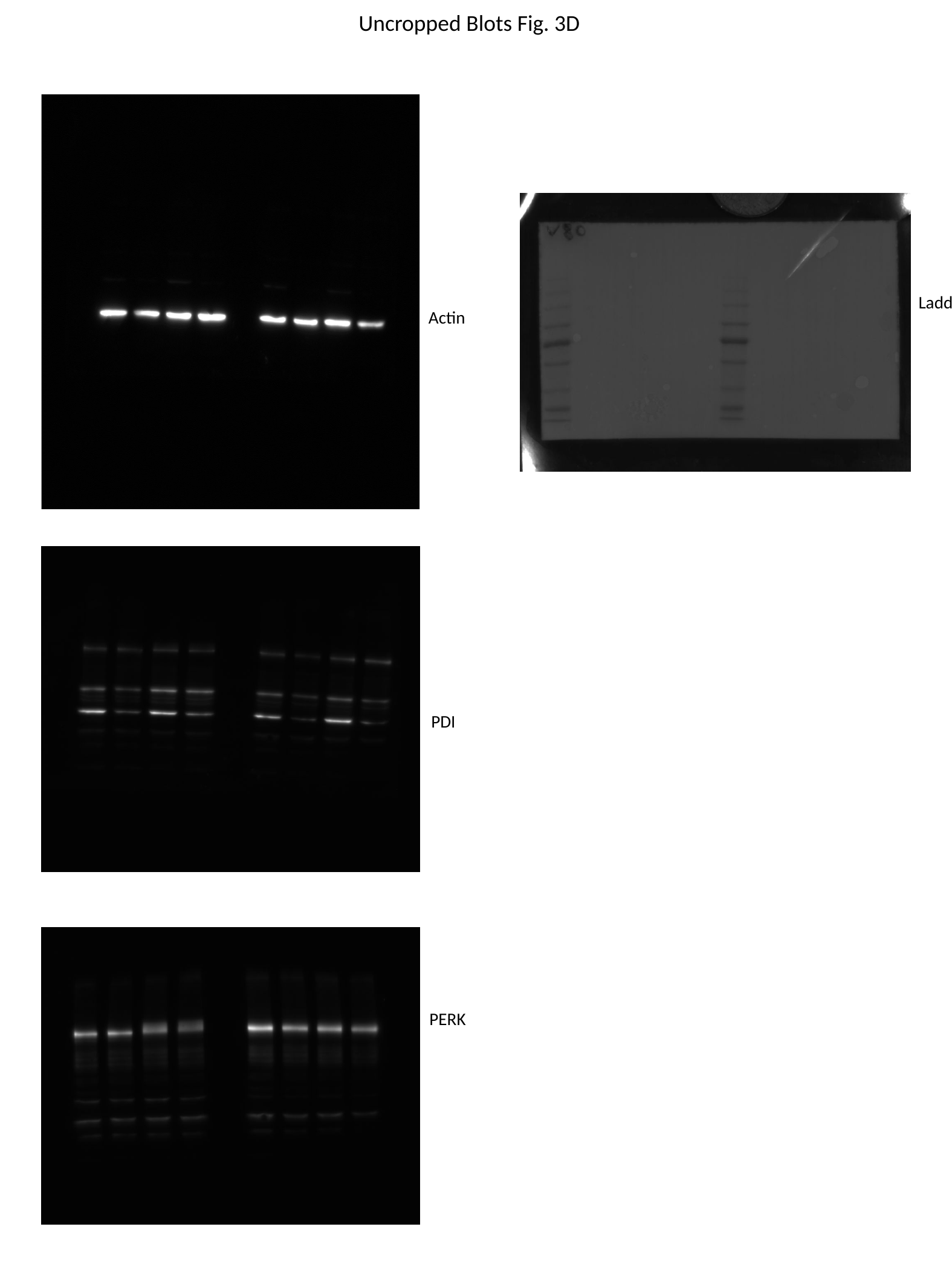

Uncropped Blots Fig. 3D
Ladder
Actin
PDI
PERK

## Slide 8
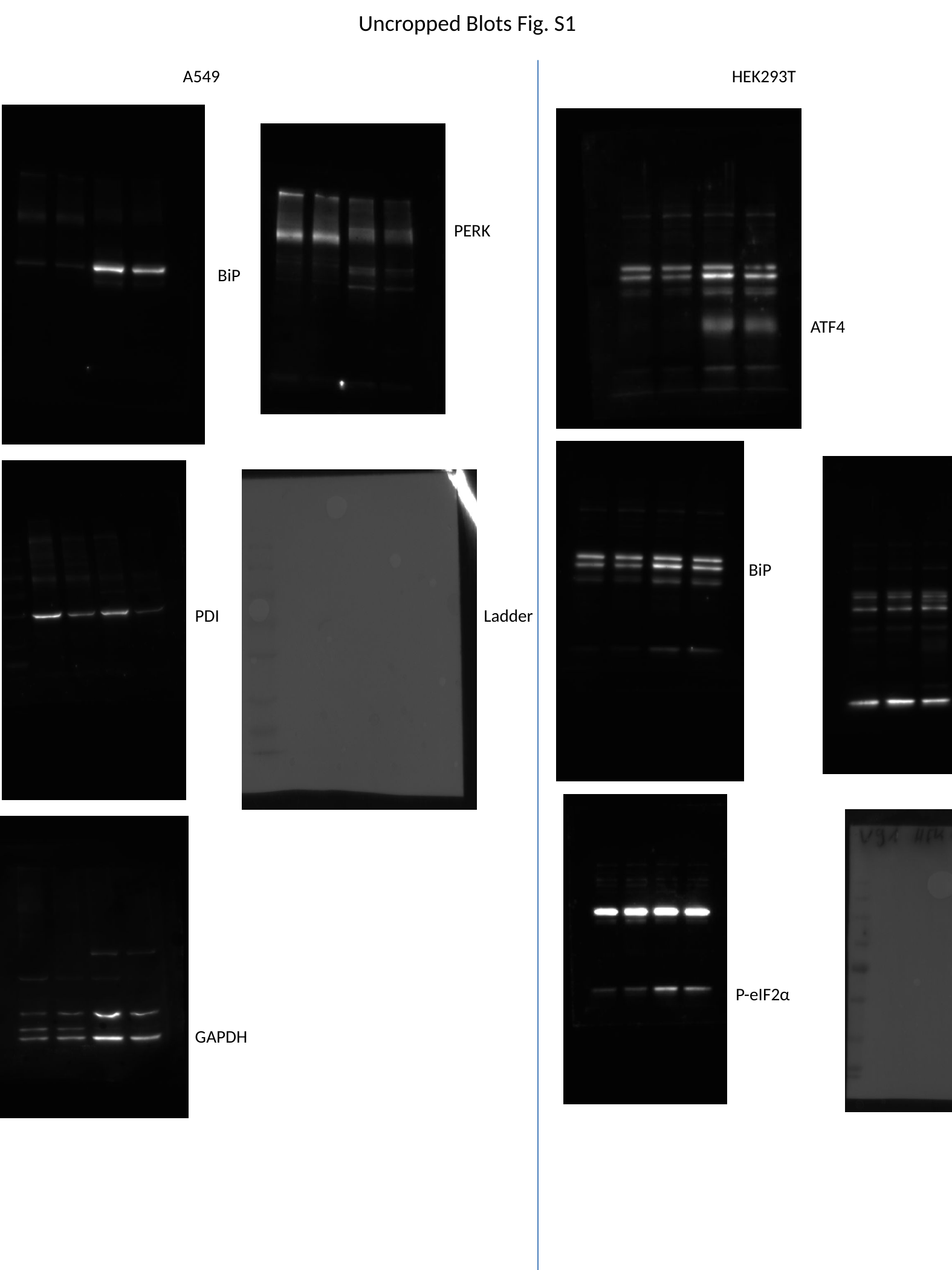

Uncropped Blots Fig. S1
A549
HEK293T
PERK
BiP
ATF4
BiP
PDI
Ladder
GAPDH
Ladder
P-eIF2α
GAPDH
